# Acute introduction of phosphoserine-129 α-synuclein induces severe swelling of mitochondria at lamprey synapses

**DOI:** 10.17912/micropub.biology.001206

**Published:** 2024-05-23

**Authors:** Caroline V. Casiano Rivera, Jaqulin N. Wallace, Gia E. Fisher, Jennifer R. Morgan

**Affiliations:** 1 Eugene Bell Center for Regenerative Biology and Tissue Engineering, Marine Biological Laboratory, Woods Hole, Massachusetts, United States; 2 Biological Sciences Division, The University of Chicago

## Abstract

Abnormal synaptic aggregation of α-synuclein is linked to cognitive deficits in Parkinson’s disease (PD). While the impacts of excess α-synuclein on synaptic function are well established, comparatively less is known about the effects on local mitochondria. Here, we examined morphological features of synaptic mitochondria treated with wild type (WT) or phosphoserine 129 (pS129) α-synuclein, a variant with prominent synaptic accumulation in PD. Acute introduction of pS129 α-synuclein to lamprey synapses caused an activity-dependent swelling and bursting of mitochondria, which did not occur with WT α-synuclein. These pS129-induced effects on mitochondria likely contribute to the synaptic deficits observed in PD.

**Figure 1. pS129 α-synuclein causes synaptic mitochondria to swell and burst:**
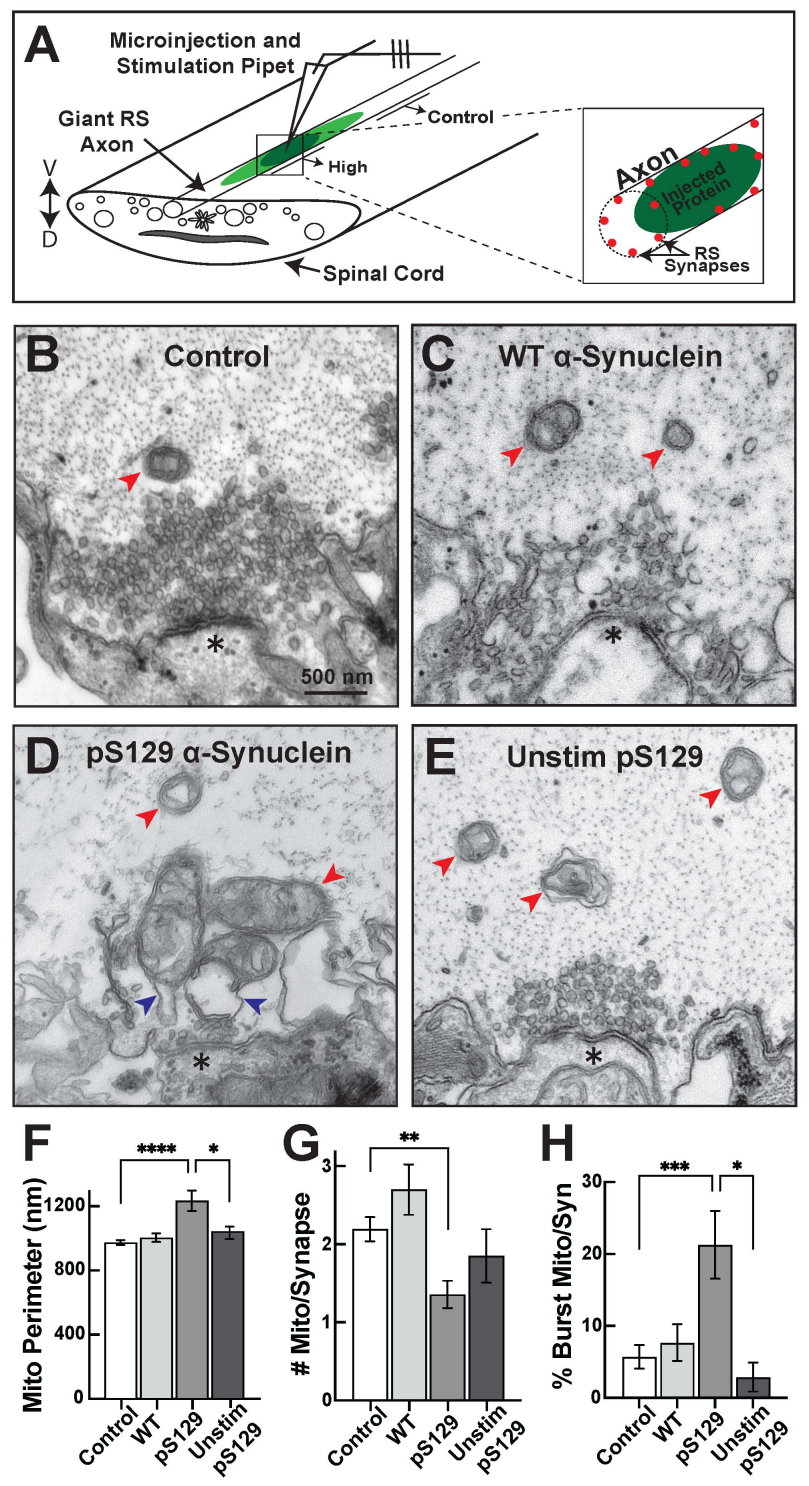
**(A)** Diagram showing the microinjection strategy for α-synuclein into lamprey giant reticulospinal (RS) axons. Inset shows how the protein is delivered to synapses. **(B)** Electron micrograph showing a control, stimulated lamprey RS synapse (20 Hz, 5 min) with representative synaptic mitochondria (red arrows). Scale bar in B applies to C-E. Asterisks indicate postsynaptic density. **(C)** Stimulated synapse treated with WT α-synuclein (10-20 μM) shows no obvious change to the morphology of mitochondria (red arrows). **(D)** In comparison, in the presence of pS129 α-synuclein (10-20 μM), synaptic mitochondria became swollen and generally dysmorphic. Blue arrows indicate burst mitochondria. **(E)** In the absence of stimulation, pS129 α-synuclein had little effect on mitochondrial morphology. **(F)** Quantification revealed a significant, activity-dependent increase in the outer perimeter of synaptic mitochondria with pS129 α-synuclein. **(G)** Stimulated synapses treated with pS129 α-synuclein had fewer mitochondria. **(H)** The percentage of burst mitochondria was also significantly higher after treatment with pS129 α-synuclein compared to all other conditions. Bars represent mean +/- standard error of the mean (SEM) from n= 31-245 mitochondria from 20-125 synapses; N=2-4 axons per condition. Asterisks indicate statistical significance by one-way ANOVA as follows: * p<0.05; ** p<0.01; *** p<0.0005; **** p<0.0001.

## Description

α-Synuclein is a synaptic vesicle-associated protein of 140 amino acids that participates in synaptic vesicle exocytosis and endocytosis under normal physiological conditions (Sulzer and Edwards, 2019; Sharma and Burre, 2022) . In synucleinopathies, such as Parkinson’s disease (PD) and Dementia with Lewy Bodies (DLB), α-synuclein aberrantly aggregates throughout neurons, including at synapses where the pathological accumulation correlates with cognitive deficits and dementia (Kramer and Schulz-Schaeffer, 2007; Schulz-Schaeffer, 2010) . Determining the impacts of excess α-synuclein at synapses is therefore an essential step toward developing strategies that can improve synapse function in PD, DLB, and other synucleinopathies. We and others have reported that acutely increasing α-synuclein levels at lamprey synapses, or overexpression at mammalian synapses, severely inhibits synaptic vesicle trafficking (Nemani et al., 2010; Scott et al., 2010; Busch et al., 2014) . At lamprey reticulospinal (RS) synapses, acute introduction of excess human α-synuclein inhibited clathrin-mediated and bulk endocytosis upon high frequency stimulation (Busch et al., 2014; Medeiros et al., 2017; Banks et al., 2020) . Selective impairment of synaptic vesicle endocytosis was also demonstrated at the mammalian calyx of Held synapse, where it was additionally shown that exocytosis was relatively unaffected (Xu et al., 2016; Eguchi et al., 2017) . Interestingly, excess phosphoserine 129 α-synuclein (pS129 α-synuclein), a post-translationally modified α-synuclein that builds up 10- to 20-fold in PD and DLB (Fujiwara et al., 2002; Anderson et al., 2006) , further impaired synaptic vesicle trafficking by inducing pronounced vesicle declustering/reclustering defects (Wallace et al., 2024) . While the impacts of excess α-synuclein on synapses are now well established, in comparison very little is known about the impacts of α-synuclein on the local mitochondria at synapses and therefore their possible influence on the synaptic phenotypes reported.

Mitochondrial ATP production at synapses occurs in an activity-dependent manner and is essential for maintaining normal synaptic function (Rangaraju et al., 2014; Rossi and Pekkurnaz, 2019) . Both endogenous and exogenous α-synuclein bind to mitochondria via interactions with cardiolipin, which is found in the inner mitochondrial membrane (Nakamura et al., 2011; Zigoneanu et al., 2012) . α-Synuclein interactions with mitochondria trigger rapid oligomerization and pore-forming activity, which can lead to neurotoxicity under pathological conditions (Ghio et al., 2019; Choi et al., 2022) . α-Synuclein overexpression also impairs mitochondrial fusion and fission dynamics in cell lines and neurons, leading to aberrant fragmentation of mitochondria (Kamp et al., 2010; Nakamura et al., 2011; Berthet et al., 2014) . In cell fractionation assays, pS129 α-synuclein isolated from the brains of PD animal models and synucleinopathy patients preferentially associated with mitochondria, whereas wild type (WT) α-synuclein was more abundant in the cytosolic/microsomal fractions (Wang et al., 2019) . Moreover, α-synuclein-rich Lewy bodies and neurites within the brains of PD patients are replete with swollen, dysmorphic mitochondria, which suggests altered ATP production or other deleterious impacts on cellular metabolism (Shahmoradian et al., 2019) . Collectively, these findings implicate α-synuclein accumulation and mitochondrial dysfunction as co-contributors in the pathogenesis of Parkinson’s disease (Nakamura, 2013; Nguyen et al., 2019; Gilmozzi et al., 2020) . However, we still don’t know how α-synuclein accumulation specifically affects the population of mitochondria localized at or near synapses, which could profoundly contribute to the synaptic dysfunction and cognitive deficits observed in PD and DLB.

One inherent challenge to addressing this problem is the small size of synaptic boutons in most vertebrate synapse models. To overcome this, we are taking advantage of the lamprey giant RS synapse, a classical vertebrate synapse model where presynapses are exceptionally large (1-2 μm diameter) and easily identifiable along the perimeter of the giant RS axons. The giant RS axons can be acutely microinjected with recombinant human α-synuclein, thereby delivering the protein directly to the giant RS synapses with or without stimulation **( Figure 1A )** , followed by ultrastructural analysis with electron microscopy (Walsh et al., 2018) . The synaptic vesicle trafficking deficits caused by acute introduction of monomeric WT and pS129 α-synuclein to lamprey synapses have already been published and collectively demonstrate severe impairment of vesicle endocytosis (Busch et al., 2014; Banks et al., 2020; Wallace et al., 2024) . Following up on these studies, we aimed here to determine the extent to which α-synuclein specifically affects the ultrastructure of mitochondria at or near synapses (within ~2-3 μm of the active zone), including any changes caused by post-translational modifications such as pS129. WT and pS129 α-synuclein were acutely injected into lamprey giant RS axons for 15-20 minutes, as previously described, followed by action potential stimulation at 20 Hz for 5 minutes before fixation and processing for electron microscopy (Busch et al., 2014; Banks et al., 2020; Wallace et al., 2024) . The final axonal concentration of α-synuclein was 10-20 μM, which is ~2-3 times the concentration of endogenous α-synuclein and commensurate with overexpression levels in PD (Singleton et al., 2003; Westphal and Chandra, 2013; Wilhelm et al., 2014) . As expected, stimulated control synapses showed normal mitochondria, defined by the presence of intact outer and inner membranes, cristae, and an electron dense matrix **( Figure 1B ).** Treatment with WT α-synuclein showed no striking changes in the ultrastructural features of synaptic mitochondria **( Figure 1C )** . In contrast, stimulated synapses treated with pS129 α-synuclein caused synaptic mitochondria to become enlarged and severely swollen **( Figure 1D )** . In some cases, the mitochondrial swelling led to a breakage in the outer membrane of the mitochondria that caused a herniation of the inner membrane, resulting in ‘burst’ mitochondria with an abnormal morphology and lighter color due to water influx **( Figure 1D, blue arrows ).** Since the synaptic vesicle trafficking deficits caused by pS129 α-synuclein are activity-dependent (Wallace et al., 2024) , we also examined the mitochondria at unstimulated synapses treated with pS129. In the absence of stimulation, pS129 did not induce dramatic alterations in the structure of synaptic mitochondria, indicating an activity-dependent effect **( Figure 1E ).**

Next, we quantified these effects by conducting a morphometric analysis on the size and number of synaptic mitochondria per synapse in each condition. Compared to mitochondria at stimulated control synapses or those treated with WT α-synuclein, synaptic mitochondria treated with excess pS129 α-synuclein were larger in size, as shown by a significant increase in their outer perimeters, and this effect was not observed at unstimulated synapses **( Figure 1F )** (Control: 966 ± 15 nm, n=245 mitochondria from N=125 synapses, 8 axons; WT: 996 ± 27 nm, n=68 mitochondria from N=30 synapses, 2 axons; pS129: 1225 ± 64 nm, n=87 mitochondria from N=56 synapses, 4 axons; Unstim pS129: 1036 ± 39 nm, n=31 mitochondria from N=20 synapses, 2 axons; ANOVA p<0.0001) **.** Hence, pS129 α-synuclein induced mitochondrial swelling in an activity-dependent manner, leading to abnormal mitochondrial morphologies. In addition, the total number of synaptic mitochondria per synapse was significantly decreased with pS129 α-synuclein, compared to control and WT α-synuclein-treated synapses **( Figure 1G )** (Control: 2.2 ± 0.16 mitochondria/synapse, n=245 mitochondria from N=125 synapses, 8 axons; WT: 2.7 ± 0.3 mitochondria/synapse, n=68 mitochondria from N=30 synapses, 2 axons; pS129: 1.4 ± 0.2 mitochondria/synapse, n=87 mitochondria form N=56 synapses, 4 axons; Unstim pS129: 1.9 ± 0.3 mitochondria/synapse, n=31 mitochondria from N=20 synapses, 2 axons; ANOVA, p=0.0007) **.** Finally, the percentage of burst mitochondria was significantly greater at stimulated synapses treated with pS129 α-synuclein, but not at unstimulated pS129-treated synapses **( Figure 1H )** (Control: 5.7 ± 1.7 % burst mitochondria/synapse, n=245 mitochondria from N=125 synapses, 8 axons; WT: 7.7 ± 2.6 % burst mitochondria/synapse, n=68 mitochondria from N=30 synapses, 2 axons; pS129: 21.3 ± 4.7 % burst mitochondria/synapse, n=87 mitochondria from N=56 synapses, 4 axons; Unstim pS129: 2.9 ± 2.0 % burst mitochondria/synapse, n=31 mitochondria from N=20 synapses, 2 axons; ANOVA, p=0.0002). Thus, acute introduction of pS129 α-synuclein, but not WT α-synuclein, induces rapid, activity-dependent swelling of synaptic mitochondria that likely contributes to their bursting.

Taken together, these data implicate mitochondrial dysfunction as a possible contributor to the synaptic deficits caused by excess pS129 α-synuclein, which we recently reported (Wallace et al., 2024) . The working model is that pS129 α-synuclein binds avidly to synaptic mitochondria, causing them to swell and making them more susceptible to bursting, which could explain the decreased numbers of mitochondria at synapses. We do not yet understand the mechanism underlying the activity-dependence of these effects. Nor can we rule out any impacts of excess pS129 α-synuclein on mitochondrial dynamics and trafficking, which will require live imaging. However, the rapid changes in the mitochondrial morphology caused by elevated pS129 α-synuclein levels suggests profound impacts on mitochondrial functions. At synapses, any downstream impacts on ATP production and/or calcium buffering would impair synaptic vesicle trafficking and ultimately neurotransmission. Recent studies have established that pS129 α-synuclein is generated at synapses under physiological conditions, an activity-dependent event that regulates synaptic transmission, vesicle clustering, and interactions with other presynaptic proteins (Parra-Rivas et al., 2023; Ramalingam et al., 2023) . Our results now suggest that dysregulation of this process in ways that aberrantly increase pS129 levels at synapses, as occurs in synucleinopathies (Colom-Cadena et al., 2017) , may also induce mitochondrial dysfunction, which would consequently impact synaptic function. However, any mechanisms directly linking the mitochondrial dysfunction to synaptic deficits are still unknown, and additional studies will be needed to obtain a comprehensive understanding of the downstream effects of the dysmorphic mitochondria in disease states. Nonetheless, the data presented here provide further insight into the disruption of cellular dynamics caused by pS129 α-synuclein and how it may impact synaptic pathologies in PD and DLB.

## Methods

***Spinal cord dissections and microinjections*** . All vertebrate animal procedures were approved by the Marine Biological Laboratory’s Institutional Animal Care and Use Committee. Acute perturbations of lamprey RS axons were performed as previously described (Busch et al., 2014; Banks et al., 2020; Wallace et al., 2024) . Late-stage larval lampreys ( *Petromyzon marinus* , 10-13 cm; M/F) were anesthetized in 0.1g/L tricaine methanesulfonate (Syncaine; Syndel, Ferndale, WA). Then, segments of spinal cords (2-3 cm) were dissected and pinned ventral side up in a Sylgard-lined petri dish (Ellsworth Adhesives; Germantown, WI) and submerged in oxygenated lamprey ringer (100 mM NaCl, 2.1 mM KCl, 1.8 mM MgCl _2_ , 4 mM sucrose, 2 mM HEPES, 0.5 mM L-glutamine, 2.6 mM CaCl _2_ ; pH 7.4). Prior to injections, WT and pS129 α-synuclein were dialyzed into lamprey internal solution (180 mM KCl, 10 mM HEPES K+; pH 7.4) to a concentration of 185-195 μM. The protein was then loaded into glass microelectrodes and injected directly into axons using small puffs of nitrogen (5-10 ms; 40 psi, 0.2 Hz) delivered by a Toohey Spritzer. In order to estimate the final axonal concentration, α-synuclein was co-injected with a fluorescent dye of comparable molecular weight (0.1 mM FITC dextran, 10 kDa; Thermo Fisher). Following injection and diffusion in the axon, α-synuclein proteins were diluted 1/10th to 1/20th of their starting concentration resulting in an estimated final concentration of 10-20 μM **(see Figure 1A )** . Injected axons were injected with small pulses of current (1 ms, 20-90 nA) in order to induce action potentials at 20 Hz for 5 minutes unless otherwise stated. For the unstimulated pS129 condition, axons were impaled with microelectrodes and injected for comparable amounts of time (15-20 minutes) with similar amounts of α-synuclein, the only difference being the lack of stimulation. Spinal cords were immediately fixed in 3% glutaraldehyde, 2% paraformaldehyde in a 0.1M sodium cacodylate buffer, pH 7.4 for at least 3 hours at room temperature and then overnight at 4°C.

***Electron microscopy*** . Fixed spinal cords were processed and embedded for standard EM, sectioned at ~70 nm, and placed on copper Formvar slot grids (EM Sciences; Hatfield, PA) as described (Walsh et al., 2018; Wallace et al., 2024) . Electron micrographs were obtained at 37,000X magnification using a JEOL JEM – 200CX electron microscope (JEOL; Peabody, MA) using a Hamamatsu C8484-05G Digital CCD side mount camera (Hamamatsu Photonics; Shizuoka, Japan). Synapse images were analyzed from 2 regions: control (>400 μm from injection site); and high concentration (<140 μm from injection site) **(see Figure 1A )** . Morphometric analyses were performed on all mitochondria visible within 2-3 μm of the active zone by a researcher blinded to the experimental conditions. Mitochondrial morphologies were obtained from: n=125 control synapses from N=8 replicates/axons; n=30 stimulated WT α-synuclein synapses from N=2 replicates/axons; n= 56 stimulated pS129 α-synuclein synapses from N=4 replicates/axons; and n=20 unstimulated pS129 α-synuclein synapses from N=2 replicates/axons. Measurements included the outer perimeter of synaptic mitochondria (in nm), number of mitochondria per synapse, and the percentage of burst mitochondria per synapse. Burst mitochondria were considered as those dysmorphic mitochondria containing a break in the outer membrane and herniation of the inner membrane. All measurements were performed using FIJI software, version 2.9.0.

***Statistical analyses*** . For all datasets, outlier tests were performed using the ROUT method. Only a few outliers were identified in the mitochondrial perimeter dataset, as follows: (Control n=9; WT n=2, pS129 n=13, pS129 unstim n=0), however they were not removed from the analysis. No outliers were identified in the other datasets. Variances were similar between WT and pS129 groups (F test; p>0.05). All graphing and statistical analyses were performed in GraphPad Prism 10. Data are reported as +/- standard error of the mean (SEM) per section per synapse. An ordinary one-way ANOVA test was performed for all datasets, followed by multiple *post hoc* comparisons for all conditions. Significance threshold was established using a p-value p<0.05. The results of the statistical analyses, including p-values, are reported in **Figure 1** and the Description section.

## Reagents

**Table:** 

**PROTEIN**	**SPECIES**	**EXPRESSION VECTOR**	**HOST**	**REFERENCES**
α-Synuclein	Human	pET28b (rPeptide)	*E. coli* BL21 (DE3)	*Medeiros* et al., 2017; *Banks et al., 2020*
pS129 α-Synuclein	Human	Proprietary information (Proteos, Inc.)	*E. coli*	*Wallace et al., 2024*
